# In vitro assessment of antibacterial and antiviral activity of three copper products after 200 rounds of simulated use

**DOI:** 10.1007/s10534-023-00572-z

**Published:** 2023-12-22

**Authors:** Marthe K. Charles, Teresa C. Williams, Davood Nakhaie, Tracey Woznow, Billie Velapatino, Ana C. Lorenzo-Leal, Horacio Bach, Elizabeth A. Bryce, Edouard Asselin

**Affiliations:** 1 Division of Medical Microbiology and Infection Prevention and Control, Vancouver Coastal Health, Vancouver, BC Canada; 2 Department of Pathology and Laboratory Medicine, University of British Columbia, Vancouver, BC Canada; 3 Department of Materials Engineering, University of British Columbia, Vancouver, BC Canada; 4 Vancouver, Canada; 5 Division of Infectious Diseases, Department of Medicine, University of British Columbia, Vancouver, BC Canada

**Keywords:** Copper, Self-sanitizing surfaces, Antimicrobial activity, Coronavirus, Norovirus, Disinfectant, Bacterial pathogens

## Abstract

Copper has well-documented antibacterial effects but few have evaluated it after prolonged use and against bacteria and viruses. Coupons from three copper formulations (solid, thermal coating, and decal applications) and carbon steel controls were subjected to 200 rounds simulated cleaning using a Wiperator™ and either an accelerated hydrogen peroxide, quaternary ammonium, or artificial sweat products. Antibacterial activity against *S. aureus* and *P. aeruginosa* was then evaluated using a modified Environmental Protection Agency protocol. Antiviral activity against coronavirus (229E) and norovirus (MNV-1) surrogates was assessed using the TCID_50_ method. Results were compared to untreated control coupons. One hour after inoculation, *S. aureus* exhibited a difference in log kill of 1.16 to 4.87 and *P. aeruginosa* a log kill difference of 3.39–5.23 (dependent upon copper product and disinfectant) compared to carbon steel. MNV-1 demonstrated an 87–99% reduction on each copper surfaces at 1 h and 99% reduction at 2 h compared to carbon steel. Similarly, coronavirus 229E exhibited a 97–99% reduction after 1 h and 90–99% after 2 h. Simulated use with artificial sweat did not hinder the antiviral nor the antibacterial activity of Cu surfaces. Self-sanitizing copper surfaces maintained antibacterial and antiviral activity after 200 rounds of simulated cleaning.

**Graphical abstract:**

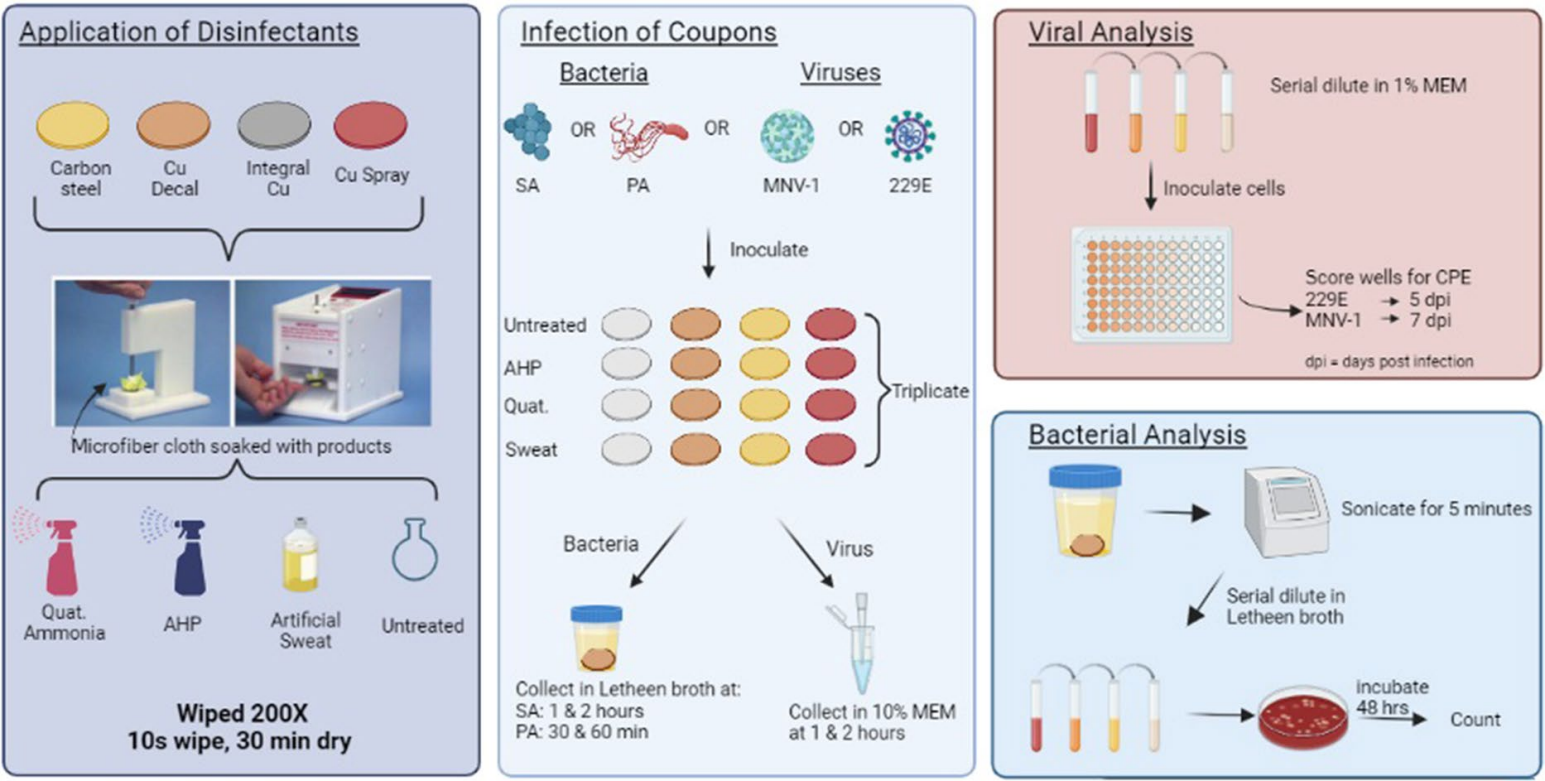

## Introduction

Copper (Cu) has a well-described role as an antimicrobial substance dating back to ancient Egypt (Borkow and Gabbay 2009). More recently, Cu and its alloys have been recognized as a mitigation measure in reducing transmission of organisms of public health concern and incorporated on highly touched surfaces (Salgado et al. 2013; Akhidime et al. 2019; Michels et al. 2015; Schmidt et al. 2016; von Dessauer et al. 2016). The precise mechanism of Cu’s antimicrobial ability is unclear, however, it has been proposed that released surface Cu ions induces membrane damage, production of reactive oxygen species (ROS), and DNA degradation (Salah et al. 2021; Grass et al. 2011). In support of these mechanisms, multiple studies have reported using chelating agents to mitigate the release of Cu ions and quenchers to inhibit ROS production reducing the antimicrobial efficacy of Cu (Warnes and Keevil 2013; van Doremalen et al. 2020).

The U.S. Environmental Protection Agency (EPA) created standards to measure the antimicrobial activity of Cu and then added an amended registration for viruses during the COVID-19 pandemic. The EPA copper antiviral claim requires a 95.6% Cu content and should eliminate 99.9% of viruses (such as SARS-CoV-2) within 2 h (EPA 2021). During the SARS-CoV-2 pandemic, increased interest for Cu surfaces in public settings was observed.

Evidence linking Cu surfaces to reduced healthcare-acquired infections has been limited in terms of study duration (Aillón-García et al. 2023). In addition, research has primarily focused on bacterial pathogens using Cu samples exposed to disinfectants and cleaning cloths not representative of those used in public venues and/or subjected samples to simulated use for relatively short periods (Bryce et al. 2020; ISO 2022; EPA 2008). There has been some promising research demonstrating that Cu has excellent antiviral activity but studies are scarce and limited in terms of duration of simulated use with the appropriate disinfectants (Warnes and Keevil 2013; van Doremalen et al. 2020; Warnes et al. 2015; Glover et al. 2022; Glass et al. 2022; Mertens et al. 2022).

This present study aimed to assess the long-term antimicrobial efficacy after simulated use of three types of commercially available Cu products; (1) a Cu alloy, (2) a thermal Cu spray-on coating and (3) a Cu-containing decal, that were compared to yellow painted carbon steel (CS) as a control. Longer duration of use was simulated by subjecting all products to 200 rounds of cleaning and disinfection using hospital-grade disinfectants (also used in public transportation). In addition, the effect of sweat on the antimicrobial properties of Cu to mimic in situ scenarios of surfaces being continuously touched by hands in the absence of disinfectants was evaluated.

## Materials and methods

### Sample preparation and simulated use

New samples of the three Cu products—1) a Cu alloy formulation (80% Cu, 20% Ni); 2) a thermal spray-on Cu coating (80% Cu, 20% Ni-Zn), 3) a Cu-containing decal (91.3% Cu)] and yellow painted CS (used as high visibility stanchions in public settings) were cut into 25 mm diameter round sections (coupons). The coupons were then subjected to 200 rounds of Wiperator™ (FiltaFlex Ltd, Ontario) treatment (ASTM-E2197 2018) using microfiber cloths soaked in disinfectant or non-stabilized artificial sweat BZ320 (Biochemazone, Ontario). Disinfectants used were selected from the in situ parallel transit study (unpublished) and included: ES65H Hydrogen Peroxide (AHP) (EnviroSolutions, Ontario) and Buckeye E23 quaternary ammonium (QA) (Maryland, USA). The Wiperator™ procedure consisted of 10 s of wiping with soaked cloths, followed by air drying (30 min), repeated 200 times for each coupon.

### Inoculation of metal coupons with bacterial strains

Following Wiperator™ treatment, coupons were cleaned, disinfected and dried as described in the EPA protocol. *Staphylococcus aureus* ATCC 29213 (SA) and *Pseudomonas aeruginosa *ATCC 27853 (PA) were inoculated onto test surfaces (in triplicate) and subsequently sub-cultured and enumerated on 5% Sheep’s Blood Agar (BAP) using a modified EPA protocol as previously described (Bryce et al. 2020).

### Inoculation of metal coupons with viral strains

Murine Norovirus 1 (MNV-1) (ATCC VR-1937) and human coronavirus (229E) (ATCC VR-740) viral stocks were prepared and titrated in RAW 264.7 (ATCC TIB-71) and MRC-5 (ATCC CCL-171) cells, respectively. Viral stocks were prepared as previously described at a multiplicity of infection of 0.1 and incubated for 5–7 days (Warnes and Keevil 2013; Warnes et al. 2015). Virus inoculum was prepared in an organic matrix, consisting of 90:10 ratio of artificial sweat: MEM media at 1 × 10^6^TCID50/mL (Glover et al. 2022)and 20 µL was inoculated on test surfaces (in triplicate) as per ASTM E2197 and modified EPA recommendations (Bryce et al. 2020; ASTM-E2197 2018). Viruses were removed from the coupons using 2 × 490 µL (1:50 dilution) washes of cell media and frozen at -80 °C until used for viral quantification.

### Viral quantification

Suspensions of MRC-5 cells (1 × 10^5^/well) or RAW 264.7 cells (3 × 10^4^/well) were seeded into 96-well plates and left overnight at 37 °C (Thackray et al. 2007). Media was discarded and pre-prepared serial dilutions of viruses in 1% MEM was added to the 96-well plates. Plates containing the 229E virus were incubated for 5 days at 35 °C and 5% CO_2_. MNV-1 plates were incubated for 7 days at 37 °C and 5% CO_2_. Cytopathic effects (CPE) were evaluated visually and the TCID_50_/mL was calculated using the Karber-Spearman method (Lei et al. 2021).

### Statistical analysis

Data were expressed as mean ± standard deviation (sd) from experiments done in triplicate. Differences between time points and conditions were calculated using two-way ANOVA with Tukey’s multiple comparisons tests. Statistical significance was set as *p < 0.05, **p < 0.01, ***p < 0.001, ****p < 0.0001. Data were analyzed using GraphPad Prism version 9.1.0 (GraphPad Software, San Diego CA).

## Results and discussion

### Antiviral activity

Few articles have investigated how long-term cleaning with disinfectants or wiping with artificial sweat impact the antimicrobial efficacy of Cu alloys against both bacteria and viruses under laboratory conditions comparable to those used in the field (Bryce et al. 2020; Glover et al. 2022). Three Health Canada approved Cu formulations and control were subjected to 200 rounds of simulated cleaning and disinfection. These conditions were selected to represent more than one year of weekly cleaning of public transit vehicles. Additionally, surfaces were subjected to mechanical rubbing with sweat to emulate regular skin contact in the absence of cleaning. CS was used as the comparator (using both treated and untreated controls) as painted yellow metal stanchions are the standard to assist the visually impaired.

**Fig. 1:**
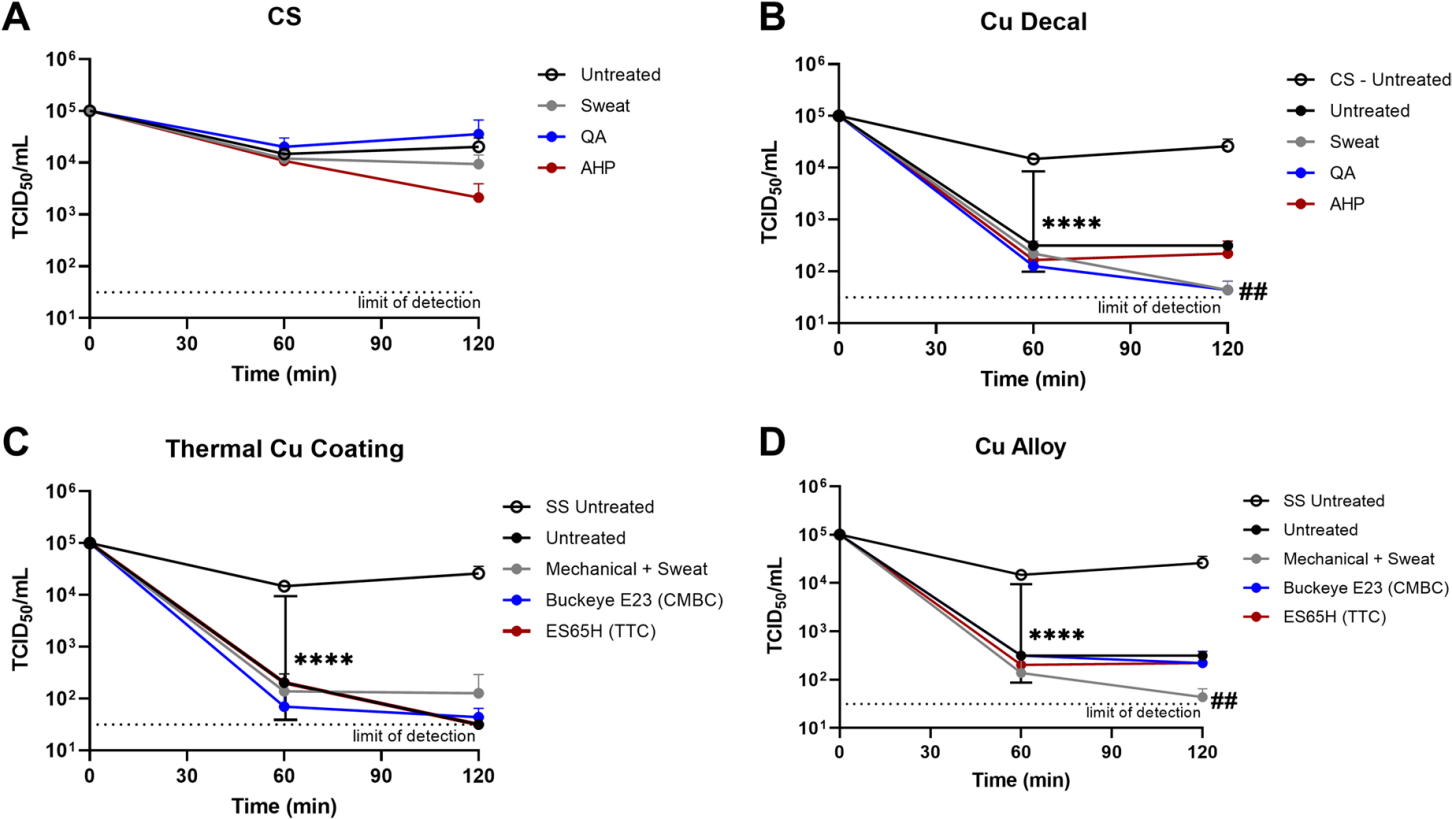
Persistence of hCoV-229E after 1-year simulated use with disinfectants and artificial sweat compared to untreated Cu coupons**.** 20 µL of 229E was applied to 4.9 cm^2^ **A** CS or **B–D** three Cu formulation coupons pre-treated with 1-year simulated cleaning or wiping with artificial sweat, QA, or AHP disinfectant and collected after 60 and 120 min. Treatments were compared to untreated control at each time-point to determine antiviral efficacy. Data is representative of triplicates, with error bars showing ± s.d. ****p > 0.0001 compared to CS untreated. ##p > 0.01 compared to the untreated condition

CS displayed no antiviral ability against hCoV-229E with either disinfectant or sweat treatment after 200 rounds of simulated use (Fig. 1a). All 3 Cu formulations showed a significant reduction in 229E viral titres after 1 h (p < 0.0001) (Fig. 1b). All Cu products maintained their antiviral activity after long-term exposure to disinfectants and sweat. After 2 h, treatment with the QA or artificial sweat completely inactivated 229E on the decal Cu product and was significantly more effective at inactivating 229E than the untreated control (p = 0.0065). All Wiperator™ treatments had the same inactivation efficacy on the Cu thermal coating application for 229E after 2 h. Finally, 200 rounds of simulated use with sweat enhanced the antiviral abilities of the alloy Cu product compared to the untreated control (p = 0.0065).

Artificial sweat was incorporated in the virus infection media at a ratio of 90:10 as per of Glover et al. to simulate real-life viral/cell interactions as closely as possible (Glover et al. 2022). In addition, it was used to wipe coupons to assess the effect of sweat exposure on metal surfaces over time. The presence of Cl^−^ at a pH of 6.5 thermodynamically favors the formation of CuCl_2_^−^ complexes and likely increased Cu ion release from the coupons. This would explain why Wiperator™ treatment with artificial sweat enhance the antiviral abilities of Cu against 229E.

**Fig. 2:**
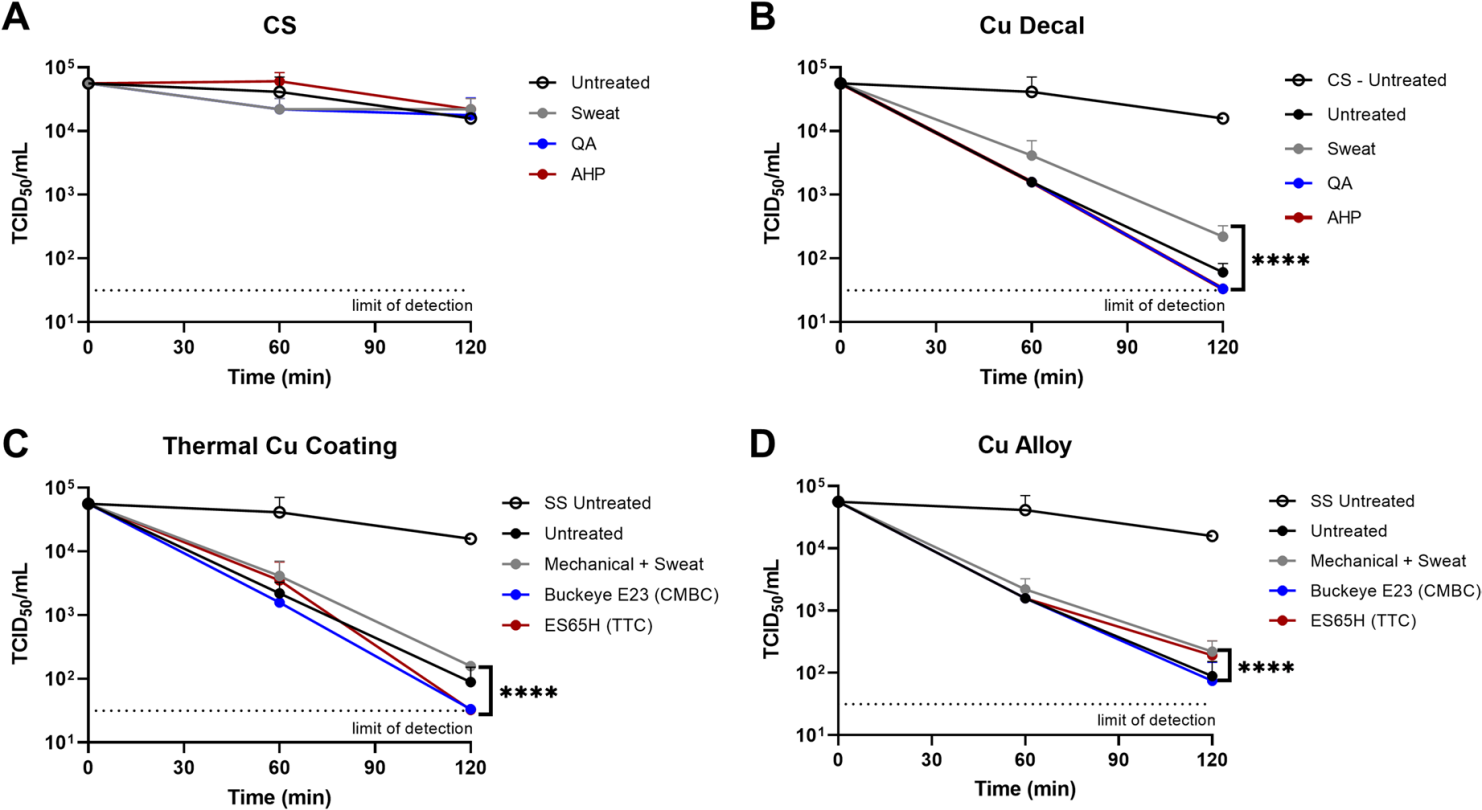
Persistence of MNV-1 after 1-year simulated use with disinfectants and sweat compared to untreated Cu coupons. 20 µL of MNV1 was applied to 4.9 cm2 **A** CS or **B**–**D** three Cu formulation coupons pre-treated with 1-year simulated cleaning or wiping with artificial sweat, QA, or AHP disinfectant and collected after 60 and 120 min. Treatments were compared to untreated control at each time-point to determine antiviral efficacy. Data is representative of triplicates, with error bars showing ± SD ****p > 0.0001 compared to the CS control

Norovirus has long been a pathogen notorious for its resistance to routine disinfectants, and its ability to survive on surfaces for many days (Mertens et al. 2022). It is a frequent cause of gastrointestinal outbreaks in public venues and healthcare facilities, and for these reasons was felt to be a most relevant entity to evaluate. As previously noted with 229E, CS showed no antiviral ability against MNV1 with any Wiperator™ disinfectant treatment (Fig.2a). Viral inactivation kinetics were similar for all three Cu formulations (Fig. 2b), regardless of the Wiperator™ treatments, when compared to the untreated control. MNV-1 titres were significantly lower on Cu surfaces than on CS control after 2 h (p < 0.0001).

During the COVID-19 pandemic, the EPA amended its antimicrobial registration to include Cu but stipulated that products must contain at least 95.6% Cu content and eliminate 99.9% of viruses within 2 h of contact. None of the Cu products evaluated in this study met the EPA Cu content antiviral criteria. However, they were still assessed as they are commercially available Cu products. Despite the lower Cu content of the products than that required by the EPA, the findings of reduced 229E and MNV-1 viral loads of between 97.2 and 99.7% in a media with simulated sweat demonstrates great promise for Cu as a long-term antiviral strategy when incorporated into high-touch surfaces.

Cu is used extensively as a component of jewelry, coins, pesticides, medical devices (e.g. intrauterine devices, dental implants), antimicrobials (copper sulfate) and yet reports of hypersensitivity are low. Studies using Cu coins, paper clips and thread left in artificial sweat for 24 h found concentrations of 0.01% Cu in solution, deemed to be too low to produce allergic reactions (Fage et al. 2014). Overall, the use of Copper onto high-touch surfaces seems to be an acceptable and safe option for product approved by the U.S. environmental protection agency or the Health Canada pest management regulatory agency.

### Bacterial results

All Cu products met sanitizer claims against PA after 1 h compared to the untreated Cu products, and antibacterial activity was not adversely affected by disinfectants or simulated sweat. Synergy with specific Cu formulations and disinfectants was observed, which has been previously noted (Bryce et al. 2020). In this study, at 30 min this synergy was observed with the Cu thermal coating product with the QA cleaner with a log10 difference of 4.92 (Table1) and the decal product with the AHP with a log10 difference of 5.31 (Table 1). Artificial sweat enhanced all 3 Cu formulations with log10 differences of 3.47–4.9 at 30 min.

**Table 1:** Biocidal activity of Cu formulations treated with 200 rounds of simulated use with disinfectants and sweat compared to control

Product carriers	Cu Decal				Thermal Cu Coating				Cu Alloy				CS	
Wiperator™ treatment	Mean	SD	% red	Log10 diff	Mean	SD	% red	Log10 diff	Mean	SD	% red	Log10 diff	Mean	SD
*Pseudomonas* *aeruginosa* (ATCC 27853) Inoculum: 3.8 × 10^7^ CFU/mL 0.5 h														
AHP	0.00E + 00	0.00E + 00	99.99	5.31	3.33E + 02	1.44E + 02	99.69	2.51	2.67E + 03	1.89E + 03	98.12	1.73	1.15E + 05	7.18E + 04
QA	1.04E + 03	0.00E + 00	99.83	2.78	2.50E + 02	4.33E + 02	99.99	4.92	7.01E + 03	4.25E + 03	98.69	1.88	4.91E + 05	1.23E + 05
Sweat	8.33E + 01	1.44E + 02	99.99	4.90	8.33E + 01	1.44E + 02	99.99	4.90	1.13E + 03	1.19E + 03	99.97	3.47	3.66E + 05	2.56E + 05
Untreated	5.07E + 03	4.79E + 03	99.46	2.27	1.08E + 03	1.46E + 03	99.98	3.69	1.71E + 03	1.90E + 03	99.97	3.50	5.43E + 05	3.65E + 05
*Pseudomonas* *aeruginosa* (ATCC 27853) Inoculum: 3.8 × 10^7^ CFU/mL 1 h														
AHP	1.67E + 02	2.89E + 02	99.99*	4.07*	0.00E + 00	0.00E + 00	99.99*	5.07*	1.67E + 02	1.44E + 02	99.94*	3.27*	6.75E + 04	4.30E + 04
QA	0.00E + 00	0.00E + 00	99.99*	5.15*	0.00E + 00	0.00E + 00	99.99*	5.15*	0.00E + 00	0.00E + 00	99.99*	5.15*	8.25E + 04	4.77E + 04
Sweat	8.33E + 01	1.44E + 02	99.99*	4.45*	0.00E + 00	0.00E + 00	99.99*	5.35*	3.33E + 02	2.89E + 02	99.95*	3.35*	1.15E + 05	3.38E + 04
Untreated	8.33E + 01	1.44E + 02	99.99*	4.52*	8.33E + 01	1.44E + 02	99.99*	4.52*	0.00E + 00	0.00E + 00	99.99*	5.42*	1.53E + 05	1.09E + 05
*Staphylococcus* *aureus *(ATCC 29213) Inoculum: 4.3 × 10^7^ CFU/mL 1 h														
AHP	1.67E + 02	2.89E + 02	99.99*	5.97*	5.96E + 05	1.92E + 05	87.74	0.91	2.03E + 05	1.25E + 05	96.40	1.44	4.72E + 06	8.44E + 05
QA	1.15E + 05	3.02E + 04	99.10	2.05	1.38E + 04	1.67E + 04	99.87	2.88	1.47E + 06	7.31E + 05	76.93	0.64	7.68E + 06	6.96E + 06
Sweat	5.29E + 04	4.25E + 03	98.44	1.81	5.08E + 05	1.21E + 05	85.24	0.83	9.86E + 05	3.08E + 05	71.92	0.55	3.65E + 06	1.72E + 06
Untreated	1.25E + 04	5.20E + 03	99.44	2.25	3.12E + 05	1.86E + 05	87.55	0.90	4.41E + 05	5.85E + 04	78.87	0.68	2.28E + 06	1.05E + 06
*Staphylococcus* *aureus* (ATCC 29213) Inoculum: 4.3 × 10^7^ CFU/mL 2 h														
AHP	5.93E + 04	5.65E + 04	99.36	2.19	1.18E + 05	6.16E + 04	97.76	1.65	2.20E + 05	2.27E + 04	95.49	1.35	5.10E + 06	1.80E + 06
QA	3.50E + 04	3.02E + 04	99.80	2.71	6.67E + 02	7.64E + 02	99.99*	4.89*	3.27E + 05	1.81E + 05	94.94	1.30	5.84E + 06	1.81E + 06
Sweat	8.31E + 04	7.42E + 04	98.58	1.85	4.14E + 05	1.06E + 05	89.85	0.99	2.60E + 05	1.10E + 05	93.84	1.21	3.99E + 06	4.09E + 05
Untreated	1.00E + 05	9.09E + 04	97.34	1.58	1.76E + 05	1.46E + 05	95.05	1.31	2.69E + 05	7.92E + 04	90.86	1.04	3.79E + 06	3.54E + 06

AHP, 7.25% accelerated hydrogen peroxide; QA, 8.45% quaternary ammonium. CS, yellow paint-coated carbon steel. Percent reduction (% red) and Log10 difference (Log10 diff) compared to CS control at matching bacteria post-exposure time-point. CS control mean ± SD: 2.4 × 10^5^ ± 1.3 × 105 for PA; 4.6 × 10^6^ ± 2.3 × 106 for SA

*Considered a sanitizer (≥ 99.9% reduction, ≥ 3 Log10 reduction, after 60 min exposure) according to EPA protocol

Similarly to PA, SA demonstrated synergy with the decal Cu product and an AHP cleaner at 1 h with a log10 difference of 5.97 (Table 1). The alloy Cu product had a range of 1.04–1.35 log10 differences after 2 h of contact. As gram positive bacteria are more resistant to antibacterial effects of Cu, we observed slower kill kinetics with SA and most Cu products did not meet sanitizer claims in the presence of simulated soil. It should be remembered that the value of Cu is its *sustained and continuous* antibacterial activity and it remains a good mitigation strategy despite its lower log-kill.

As the sustained and continuous release of Cu ions is vital for its antimicrobial function, future studies should investigate the release of Cu in high-touch areas over long periods of time and how this affects its physical composition.

## Conclusion

This study demonstrates that Cu concentrations below the EPA requirement of 95.6% display significant antiviral capabilities when assessed using EPA approved enveloped and non-enveloped viruses. Further, Cu maintains its antimicrobial abilities over 200 rounds of simulated use with a Wiperator™ using two common industry-approved cleaners as well as artificial sweat.

## References

[CR1] Aillón-García P, Parga-Landa B, Guillén-Grima F (2023). Effectiveness of copper as a preventive tool in healthcare facilities. A systematic review. Am J Infect Control.

[CR2] Akhidime ID, Saubade F, Benson PS, Butler JA, Olivier S, Kelly P, Verran J, Whitehead KA (2019). The antimicrobial effect of metal substrates on food pathogens. Food Bioprod Process.

[CR3] ASTM-E2197 (2018) Standard quantitative disk carrier test method for determining bactericidal, virucidal, fungicidal, mycobactericidal, and sporicidal activities of liquid chemicals germicides. Book Stand ASTM Int West Conshohocken PA 11(8):13. 10.1520/E2197-11

[CR4] Borkow G, Gabbay J (2009). Copper, an ancient remedy returning to fight microbial, fungal and viral infections. Curr Chem Biol.

[CR5] Bryce EA, Velapatino B, Akbari Khorami H, Donnelly-Pierce T, Wong T, Dixon R, Asselin E (2020). In vitro evaluation of antimicrobial efficacy and durability of three copper surfaces used in healthcare. Biointerphases.

[CR6] EPA (2008). Antimicrobial copper alloys-group V.

[CR7] EPA (2021). EPA registers copper surfaces for residual use against coronavirus.

[CR8] Fage SW, Faurschou A, Thyssen JP (2014). Copper hypersensitivity. Contact Dermat.

[CR9] Glass A, Klinkhammer KE, Christofferson RC, Mores CN (2022). Efficacy of copper blend coatings in reducing SARS-CoV-2 contamination. Biometals.

[CR10] Glover CF, Miyake T, Wallemacq V, Harris JD, Emery J, Engel DA, McDonnell SJ, Scully JR (2022). Interrogating the effect of assay media on the rate of virus inactivation of high-touch copper surfaces: a materials science approach. Adv Mater Interfaces.

[CR11] Grass G, Rensing C, Solioz M (2011). Metallic copper as an antimicrobial surface. Appl Environ Microbiol.

[CR12] ISO (2022). Method for the evaluation of basic bactericidal activity of a non-porous surface.

[CR13] Lei C, Yang J, Hu J, Sun X (2021). On the calculation of TCID(50) for quantitation of virus infectivity. Virol Sin.

[CR14] Mertens BS, Moore MD, Jaykus LA, Velev OD (2022). Efficacy and mechanisms of copper ion-catalyzed inactivation of human norovirus. ACS Infect Dis.

[CR15] Michels HT, Keevil CW, Salgado CD, Schmidt MG (2015). From laboratory research to a clinical trial: copper alloy surfaces kill bacteria and reduce hospital-acquired infections. HERD.

[CR16] Salah I, Parkin IP, Allan E (2021). Copper as an antimicrobial agent: recent advances. RSC Adv.

[CR17] Salgado CD, Sepkowitz KA, John JF, Cantey JR, Attaway HH, Freeman KD, Sharpe PA, Michels HT, Schmidt MG (2013). Copper surfaces reduce the rate of healthcare-acquired infections in the intensive care unit. Infect Control Hosp Epidemiol.

[CR18] Schmidt MG, von Dessauer B, Benavente C, Benadof D, Cifuentes P, Elgueta A, Duran C, Navarrete MS (2016). Copper surfaces are associated with significantly lower concentrations of bacteria on selected surfaces within a pediatric intensive care unit. Am J Infect Control.

[CR19] Thackray LB, Wobus CE, Chachu KA, Liu B, Alegre ER, Henderson KS, Kelley ST, Virgin HW (2007). Murine noroviruses comprising a single genogroup exhibit biological diversity despite limited sequence divergence. J Virol.

[CR20] van Doremalen N, Bushmaker T, Morris DH, Holbrook MG, Gamble A, Williamson BN, Tamin A, Harcourt JL, Thornburg NJ, Gerber SI, Lloyd-Smith JO, de Wit E, Munster VJ (2020). Aerosol and surface stability of SARS-CoV-2 as compared with SARS-CoV-1. N Engl J Med.

[CR21] von Dessauer B, Navarrete MS, Benadof D, Benavente C, Schmidt MG (2016). Potential effectiveness of copper surfaces in reducing health care-associated Infection rates in a pediatric intensive and intermediate care unit: a nonrandomized controlled trial. Am J Infect Control.

[CR22] Warnes SL, Keevil CW (2013). Inactivation of norovirus on dry copper alloy surfaces. PLoS ONE.

[CR23] Warnes SL, Little ZR, Keevil CW (2015). Human coronavirus 229E remains infectious on common touch surface materials. mBio.

