# Author Correction: In vitro assessment of antibacterial and antiviral activity of three copper products after 200 rounds of simulated use

**DOI:** 10.1007/s10534-024-00587-0

**Published:** 2024-02-14

**Authors:** Marthe K. Charles, Teresa C. Williams, Davood Nakhaie, Tracey Woznow, Billie Velapatino, Ana C. Lorenzo-Leal, Horacio Bach, Elizabeth A. Bryce, Edouard Asselin

**Affiliations:** 1 Division of Medical Microbiology and Infection Prevention and Control, Vancouver Coastal Health, Vancouver, BC Canada; 2 Department of Pathology and Laboratory Medicine, University of British Columbia, Vancouver, BC Canada; 3 Department of Materials Engineering, University of British Columbia, Vancouver, BC Canada; 4 Vancouver, Canada; 5 Division of Infectious Diseases, Department of Medicine, University of British Columbia, Vancouver, BC Canada

**Correction to: BioMetals** 10.1007/s10534-023-00572-z

In a recent communication with one of the manufacturers, upon publication of the manuscript, the manufacturer of the thermal coated product described in the manuscript, would normally refer to its product as “thermal fabrication”. In addition, we were informed that the description of the material being composed of 80% Cu, 20% Ni–Zn is incorrect. The authors wish to amend the description of the copper product referred to as “thermal coating” (Table 1) to “thermal fabrication”. And for the concentration of each alloy to be corrected to “approximately 67% Cu and the balance Ni-Zn”.

The authors feel that particularly correcting the concentration of copper is important, considering this concentration being significantly lower than that required by the EPA.

The authors apologize for this error and for the inconvenience. This does not change the scientific conclusions of the article in any way.

